# Laparoscopic Low Anterior Resection for Rectal Cancer in a Patient With Rectal Varices Secondary to Metabolic Dysfunction-Associated Steatohepatitis-Related Cirrhosis: A Case Report

**DOI:** 10.7759/cureus.87057

**Published:** 2025-06-30

**Authors:** Takuto Yoshida, Shin Emoto, Yuka Hosokawa, Takuji Ota, Koichi Kato, Hironobu Kikuchi, Tomoaki Kawai, Yoshimasa Tokuchi, Norihiko Takahashi, Akinobu Taketomi

**Affiliations:** 1 Department of Surgery, Iwamizawa Municipal General Hospital, Iwamizawa, JPN; 2 Department of Gastroenterology, Iwamizawa Municipal General Hospital, Iwamizawa, JPN; 3 Department of Gastroenterological Surgery 1, Hokkaido University Graduate School of Medicine, Sapporo, JPN

**Keywords:** laparoscopic low anterior resection, laparoscopic surgery, liver cirrhosis, metabolic dysfunction-associated steatohepatitis-related cirrhosis, portal hypertension, rectal cancer, rectal varices

## Abstract

Rectal varices (RVs) are a known complication of portal hypertension in patients with liver cirrhosis; however, the coexistence of rectal cancer and RVs in the setting of metabolic dysfunction-associated steatohepatitis (MASH)-related cirrhosis is rare. Herein, we report the successful laparoscopic management of rectal cancer complicated by RVs in an 80-year-old woman with Child-Pugh class A cirrhosis. A colonoscopy revealed rectal adenocarcinoma with surrounding varices. Given the patient's preserved hepatic function, we performed laparoscopic low anterior resection without preoperative variceal interventions. To minimize intraoperative bleeding, early ligation of the inferior mesenteric vein, ligation of the proximal mesenteric vein supplying the varices, and clipping and division of all visible mesenteric veins within the mesorectum were carried out. Postoperatively, portal hypertension was effectively managed with diuretics, and the patient recovered without complications or recurrent bleeding. This case demonstrates a feasible surgical strategy focused on vascular control in patients with rectal cancer, RVs, and compensated cirrhosis, offering valuable insight for managing similarly complex cases.

## Introduction

Rectal varices (RVs) commonly develop as portosystemic collaterals in patients with portal hypertension, most often due to cirrhosis [1,2]. When sensitive diagnostic tools such as endoscopic ultrasound are employed, the prevalence of RVs in cirrhotic patients is reported to be as high as 50% or more [3]. While RVs themselves are relatively uncommon, the simultaneous occurrence of rectal cancer and RVs in individuals with metabolic dysfunction-associated steatohepatitis (MASH)-related cirrhosis is rare, with limited documentation in the literature. This unique combination presents considerable clinical challenges, particularly in the surgical management of rectal malignancies, due to the heightened risk of variceal bleeding and the potential impact of impaired hepatic function.

Herein, we describe a rare case of rectal cancer complicated by RVs in a patient with MASH-related cirrhosis. We detail our surgical approach and the underlying rationale for treatment decisions. In addition, we provide a brief review of the literature regarding RVs and current surgical strategies for managing rectal cancer in patients with cirrhosis.

## Case presentation

An 80-year-old woman with a history of MASH-related cirrhosis and hepatocellular carcinoma (HCC) was referred to our department for surgical evaluation of a newly diagnosed rectal cancer. Her chief complaint was the passage of bloody stool. She had previously undergone radiofrequency ablation for HCC lesions in liver segments 3 and 5, and had a history of esophageal varices managed with endoscopic variceal ligation. Laboratory investigations revealed mildly impaired hepatic function (Table 1).

**Table 1:** Laboratory findings Laboratory values obtained at the time of initial evaluation demonstrated mildly impaired hepatic function consistent with Child-Pugh class A cirrhosis and hypersplenism.

Parameter	Result	Reference range
Platelet count	8.5 × 10^4^/μL	15.8-34.8 × 10^4^/μL
Aspartate aminotransferase	54 IU/L	13-30 IU/L
Alanine aminotransferase	26 IU/L	7-23 IU/L
Total bilirubin	1.5 mg/dL	0.3-1.2 mg/dL
Albumin	3.0 g/dL	3.8-5.3 g/dL
Prothrombin time, international normalized ratio	1.09	0.90-1.10
Carcinoembryonic antigen	6.2 ng/L	<5.0 ng/mL
Carbohydrate antigen 19-9	22.0 U/mL	<37 U/mL
Alpha-fetoprotein	6.9 ng/mL	<10 ng/mL
Child-Pugh classification	Class A (score 6)	-

**Figure 1:**
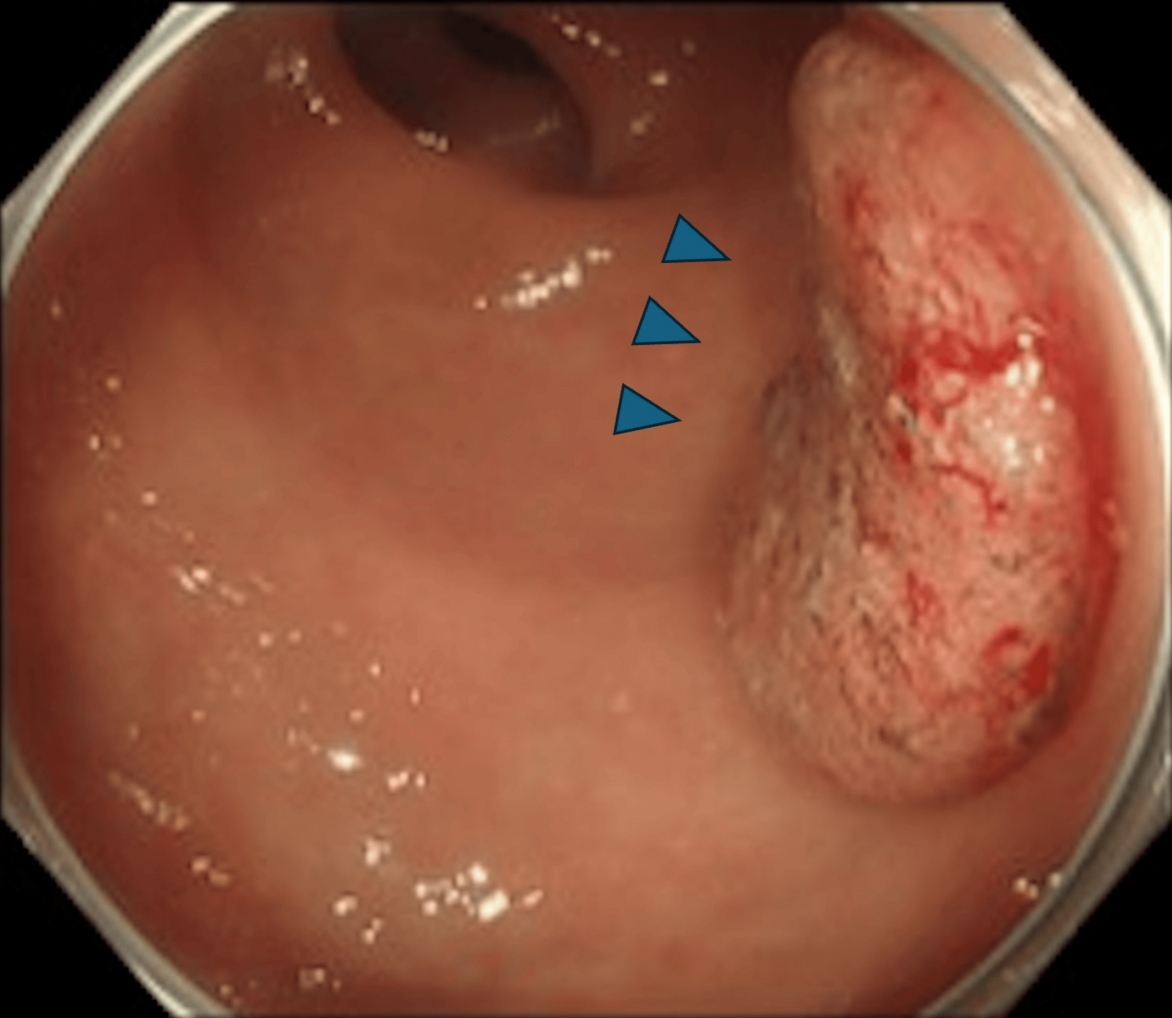
A 25-mm type 2 lesion (blue arrows) located 10 cm from the anal verge, displaying irregular surface patterns and abnormal vessels indicative of submucosal invasion.

**Figure 2:**
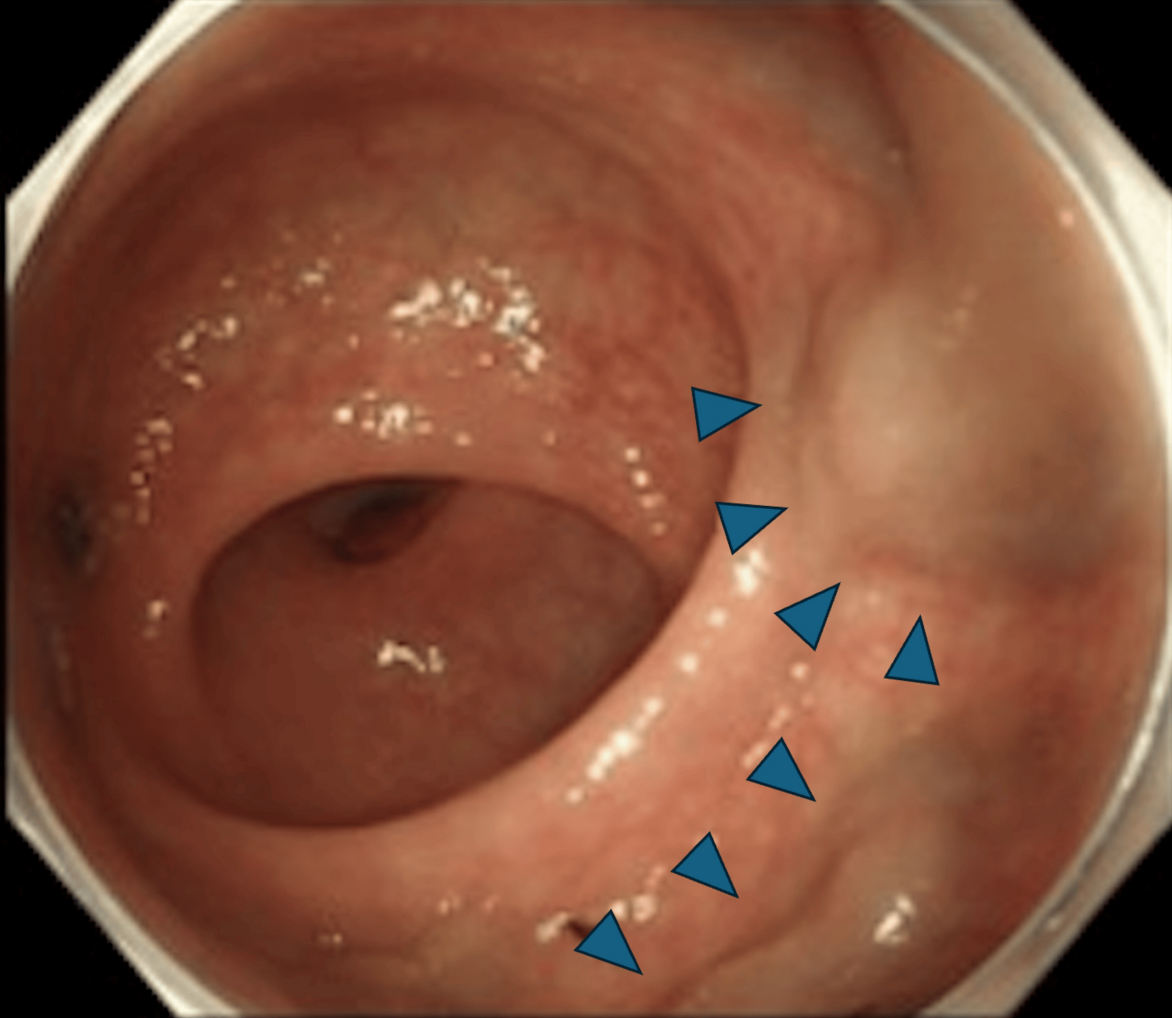
Rectal varices (blue arrows) noted close to the lesion

Colonoscopy revealed a 25-mm type 2 lesion located approximately 10 cm from the anal verge, with features strongly suggestive of submucosal invasion (Figure 1). Prominent RVs were observed surrounding the tumor (Figure 2).

**Figure 3:**
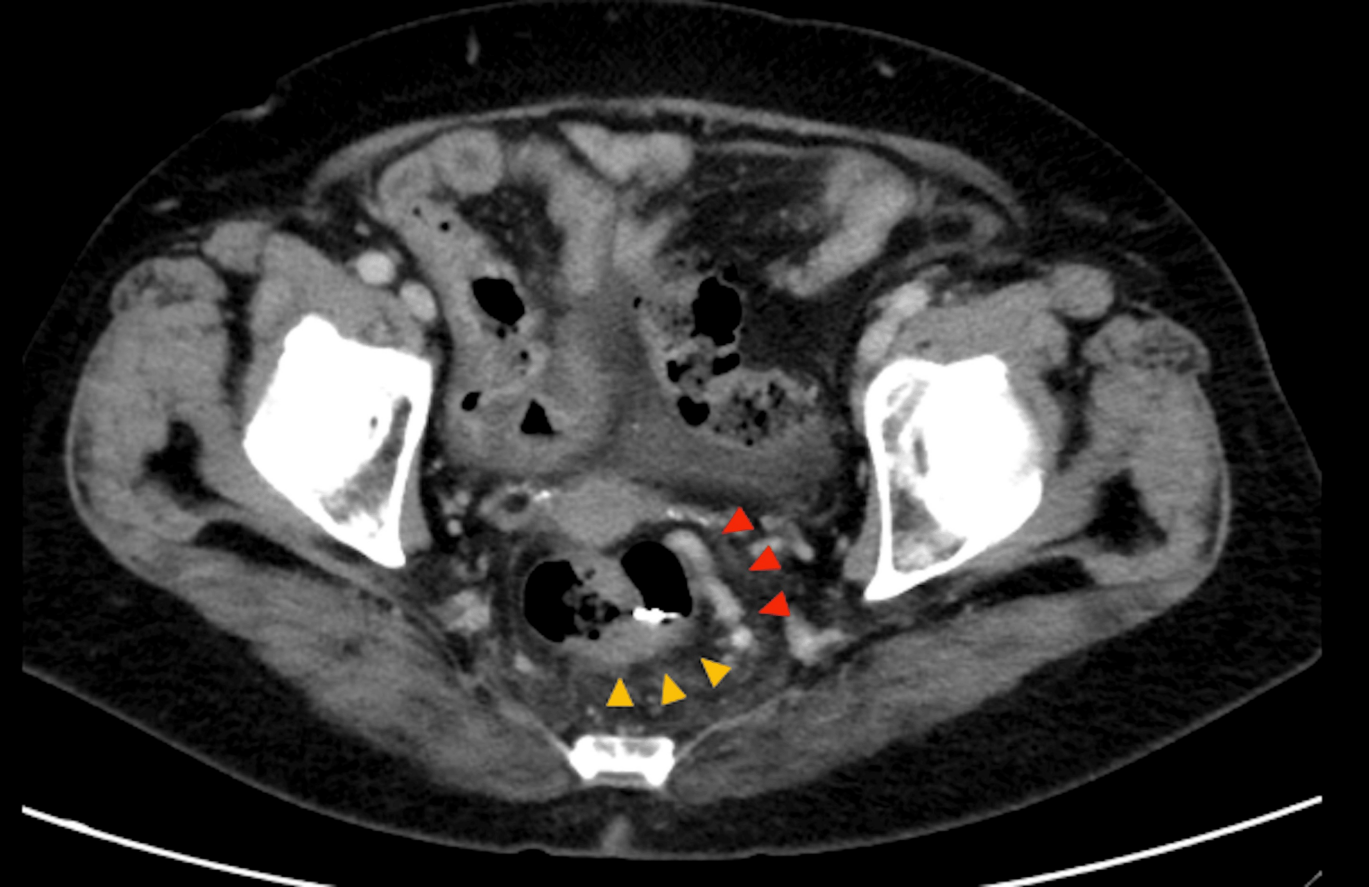
Computed tomography findings Image showing focal rectal wall thickening consistent with a primary tumor (yellow arrows), mild ascites, and prominent rectal varices (red arrows) protruding into the lumen due to portal hypertension without enlarged perirectal lymph nodes.

Biopsy confirmed a well-to-moderately differentiated adenocarcinoma. Computed tomography demonstrated rectal wall thickening consistent with a clinical T2 lesion, without evidence of lymph node metastasis. Prominent rectal varices (RVs) were also noted surrounding the tumor. Findings consistent with cirrhosis included an irregular liver surface, although no ascites were observed (Figure 3).

**Figure 4:**
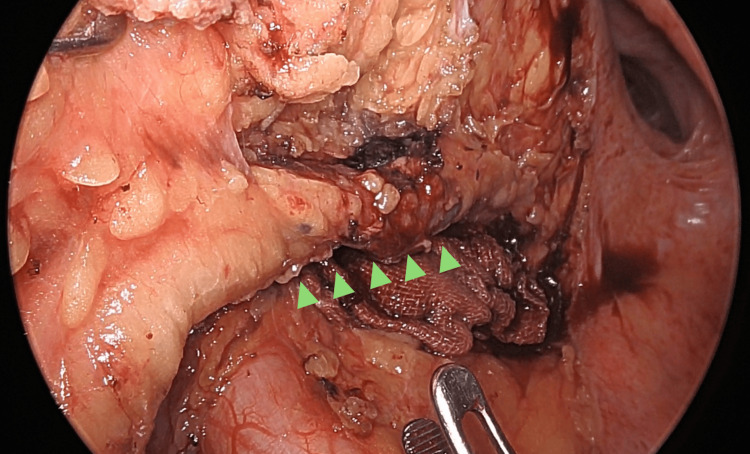
Intraoperative findings Early inferior mesenteric vein ligation followed by proximal mesenteric vein ligation (green arrows), and clipping and division of all visible mesenteric veins within the mesorectum effectively controlled variceal inflow, minimizing intraoperative bleeding risk.

Considering her preserved hepatic function and relatively low risk of bleeding, we performed laparoscopic low anterior resection and a conservative D1 lymph node dissection. Intraoperative inspection of the liver confirmed surface irregularities consistent with cirrhosis. The inferior mesenteric vein (IMV) was identified early during dissection and ligated to reduce variceal inflow from the portal system (Figure 4). The superior rectal artery was ligated shortly thereafter. However, since IMV ligation alone does not fully eliminate collateral inflow, all visible mesenteric veins within the mesorectum surrounding the planned resection site were carefully clipped and divided to minimize the risk of intraoperative bleeding. Finally, indocyanine green fluorescence imaging was used to assess tissue perfusion and confirm adequate blood flow to the planned anastomotic site.

Anastomosis was performed using a double-stapling technique with a 25-mm circular stapler (tri-EEATM, Medtronic, Minneapolis, MN, USA). Minor bleeding occurred intraoperatively but was controlled without the need for conversion to open surgery. The total operative time was 4 hours 58 minutes, with an estimated blood loss of 280 mL. Pathological examination revealed a type 2, moderately differentiated rectal adenocarcinoma invading the muscularis propria without lymph node metastasis, classified as stage I according to the Japanese classification of colorectal carcinoma [4].

The postoperative course was uneventful. Furosemide was administered on postoperative day (POD) 2 to manage portal hypertension. By POD 5, the abdominal drain was removed, and the patient resumed oral intake. Laboratory values remained stable, and the patient was discharged on POD 13. No recurrent bleeding occurred during a four-month follow-up period.

## Discussion

RVs form portosystemic collaterals arising from portal hypertension, connecting the portal venous system via the superior rectal vein to the systemic circulation via the middle and inferior rectal veins. Among patients with liver cirrhosis, the incidence of RVs is reported to be between 38% and 56% [1,2]. Major hemorrhage from these varices is relatively rare (0.5%-5%); however, once it occurs, it can become life-threatening. Unlike esophageal varices, no standard approach has been established for managing RVs. Treatment options include endoscopic band ligation, endoscopic injection sclerotherapy, transjugular intrahepatic portosystemic shunt (TIPS), and balloon-occluded retrograde transvenous obliteration. Surgical treatment is generally reserved for refractory cases, and contemporary reports on surgical management of RVs have become increasingly rare, owing to advances in endoscopic and interventional radiology techniques [3].

Although surgery is rarely a first-line approach for RVs, several reports have described successful combinations of endoscopic, radiological, and surgical interventions in complex cases. Ishiyama et al. reported a case of early rectal cancer complicated by RVs, in which preoperative endoscopic variceal ligation was performed to reduce bleeding risk, followed by successful endoscopic submucosal dissection for en bloc removal of the lesion without significant hemorrhage [5]. Similarly, De Magistris et al. described managing severe portal hypertension and rectal cancer by initially performing TIPS, followed 36 days later by laparoscopic low anterior resection without significant intraoperative bleeding or postoperative complications [6]. These experiences indicate the necessity of assessing the risk of variceal bleeding individually. Considering these factors, including preserved hepatic function (Child-Pugh class A), no prior episodes of rectal variceal bleeding, and the potential risks associated with TIPS or variceal embolization in elderly cirrhotic patients, we judged that the morbidity of additional preoperative variceal interventions outweighed the potential benefits in this case. Therefore, we proceeded directly with surgical resection while taking various intraoperative vascular control measures.

A key technical aspect of our surgical strategy was early ligation of the IMV to initially reduce inflow to the RVs. However, in this case, the surrounding varices did not demonstrate visible reduction following IMV ligation, an observation concordant with previous reports indicating that isolated IMV ligation alone cannot adequately control variceal inflow due to the presence of multiple collateral pathways in patients with cirrhosis [7]. Although we presumed a hepatofugal flow direction based on the patient's clinical history of prior esophageal variceal ligation and the typical pathophysiology of rectal varices as portosystemic collaterals, inappropriate ligation could potentially exacerbate variceal bleeding if the dominant inflow route is inaccurately identified. Thus, precise preoperative evaluation of venous flow direction and dominant collateral pathways using modalities such as Doppler ultrasonography should ideally be performed before IMV ligation to ensure the safety and efficacy of this surgical strategy. Additionally, we used a relatively low pneumoperitoneum pressure (8 mmHg) to decrease portal blood flow [8] and indocyanine green fluorescence to evaluate tissue perfusion and visually confirm varices during the procedure [9].

Postoperatively, diuretic therapy was initiated primarily to manage fluid retention and ascites, in accordance with current guidelines for cirrhotic patients [10,11]. Although diuretics alone have a limited effect on portal pressure, effective control of ascites may indirectly contribute to a reduction in portal hypertension and associated complications. Surgical removal of rectal cancer does not resolve underlying portal hypertension, and therefore, continued vigilance for variceal recurrence remains essential. Thus, regular surveillance with esophagogastroduodenoscopy and colonoscopy is necessary to monitor residual or newly formed varices over the long term.

## Conclusions

In conclusion, this case demonstrates that laparoscopic low anterior resection can be safely performed in cirrhotic patients with RVs when meticulous vascular control strategies are applied. Key measures, such as early ligation of the inferior mesenteric vein and systematic clipping and division of mesorectal veins, play a critical role in minimizing intraoperative and postoperative bleeding risks. These techniques enhance surgical safety and may improve outcomes in similarly complex cases.
